# Building organizational readiness: initial field testing of an expert-informed typology of implementation strategies

**DOI:** 10.1186/s43058-022-00257-7

**Published:** 2022-03-02

**Authors:** Sigal Vax, Marianne Farkas, Kim T. Mueser, Mari-Lynn Drainoni, Zlatka Russinova

**Affiliations:** 1 Rehabilitation Sciences Program, College of Health & Rehabilitation: Sargent College, Boston University, Boston, USA; 2 Center for Psychiatric Rehabilitation, College of Health & Rehabilitation: Sargent College, Boston University, 940 Commonwealth Ave W, Boston, MA 02215 USA; 3 Section of Infectious Diseases, Department of Medicine, Boston University School of Medicine, Boston, USA; 4 Department of Health Law Policy & Management, Boston University School of Public Health, Boston, USA

**Keywords:** Implementation strategies, Pre-implementation, Transtheoretical model, Organizational readiness for change, Evidence-based practices, Community mental health, Psychiatric rehabilitation, Psychosocial interventions, Employment services

## Abstract

**Background:**

Organizational readiness is a known barrier to implementing evidence-based practices (EBPs) in community mental health services. A robust methodology for enhancing organizational readiness for implementation (ORI) has the potential to improve implementation outcomes of EBPs and ensure better services for people with a psychiatric disability. Prior work established a framework of implementation strategies targeting ORI enhancement by asking a group of implementation experts from various fields to categorize strategies from the “Expert Recommendations for Implementing Change” (ERIC) Project into three readiness stages, consistent with the pre-action stages of the Transtheoretical Model of behavioral change: Pre-contemplation, Contemplation, and Preparation. The current study provides initial confirmation and refinement to this expert-driven typology based on community mental health field experiences.

**Methods:**

We conducted in-depth interviews with stakeholders involved in a recent EBP implementation project. Participants included staff (*n*=9) from four community mental health agencies and the implementation team who facilitated the project (*n*=3). Their pre-implementation experiences were compared with the experts’ typology to identify consistencies and discrepancies.

**Results:**

The participants’ experiences were congruent with two thirds of the strategies identified by the experts for specific ORI stages. The refinements included 12 strategies used in additional stages beyond the experts’ classification, four strategies from the ERIC list that were not included in the ORI typology, and five new strategies.

**Conclusions:**

This study provides initial confirmation and refinements to the previously published ORI typology. The results offer guidance as to how ORI could be enhanced in the community mental health field.

**Supplementary Information:**

The online version contains supplementary material available at 10.1186/s43058-022-00257-7.

Contributions to the literature

- Limited knowledge exists regarding the enhancement of organizational readiness for implementing evidence-based practices to improve their adoption in health services.
- This study followed a previous study, in which a group of implementation experts categorized implementation strategies into three stages of readiness. In this study, we compared the experiences of implementers from multiple community mental health sites to the experts’ categorization to test its relevance in the field.
- The results confirmed two thirds of the experts’ categorization and added a few variations.
- This study provides an essential step towards developing a practical approach for organizational readiness enhancement.

## Background

Numerous psychosocial interventions have been shown to be effective for supporting community participation in work, education, and independent living of people with a psychiatric disability. However, adoption of those interventions remains extremely low [1]. One of the most common reasons for the low adoption rates of evidence-based practices (EBPs) is limited organizational readiness for the desired practice change [2–5]. The concept of *Organizational readiness* is defined as “the degree to which the organization and those involved are individually and collectively primed, motivated, and capable of executing change” ( [6], p. 9). Higher readiness levels can lead to higher adoption rates of EBPs and more effective implementation processes [7, 8]. Therefore, addressing the three aspects of readiness: being primed, motivated, and prepared, across the organization—has the potential to engage more agencies in adopting EBPs successfully.

Over the last decade, research related to the effective implementation of EBPs has focused on specifying strategies to overcome barriers to successful implementation [9]. The most comprehensive compilation of such strategies was generated through the Expert Recommendations for Implementing Change (ERIC) project [10, 11], which includes 73 implementation strategies that were collected from the literature and then verified by a group of implementation experts from various health fields. Several attempts have been made to assess the relevance of these strategies to specific fields, including school-based mental health interventions [12], psychotherapy for PTSD [13], and cardiac prevention [14]. To date, such an assessment has not been conducted for psychosocial interventions in the mental health field.

In addition, although the ERIC compilation provides a comprehensive list of implementation strategies, it lacks internal organization or a framework to guide the selection of strategies at specific stages of implementation, such as pre-implementation, active implementation, sustainment, or scaling-up. The few published studies that have explored the utilization of the ERIC strategies before and during implementation described the timing of their utilization throughout the implementation process but did not provide information regarding their target outcomes [15, 16]. While these studies offer initial guidance regarding strategies that could be relevant prior to implementation, more information is needed on how these strategies may enhance specific organizational readiness benchmarks across the organization.

A recent study recruited a group of implementation experts from various fields and used a modified Delphi process to build consensus around classifying strategies from the ERIC compilation into a systematic framework that can support ORI development [17]. The experts identified strategies related to ORI enhancement from the ERIC compilation and categorized them into three readiness stages based on the Transtheoretical Model, a well-established model of behavioral change [18–20]. The three organizational readiness stages were Pre-contemplation, Contemplation, and Preparation.

The study resulted in a typology comprised of 48 pre-implementation strategies classified by ORI stages. While the expert-informed (EI) typology identified strategies that may support implementers progress through the stages of readiness development, it was constructed by experts from a variety of fields and was not specific to community mental health (CMH) services. Empirical data about the use of ORI strategies when implementing EBPs in the mental health field could help to further refine this typology.

The current study was aimed at confirming and refining the EI typology based on the field experiences of CMH stakeholders involved in a recent multi-site implementation of an EBP. A qualitative study design was used to evaluate the consistency between the experts’ classification of the ERIC strategies into the ORI stages, as constructed in the previous study, and the utilization of readiness-related strategies reported by the participants in the current study.

## Methods

### Study design

We conducted a qualitative exploratory study using in-depth interviews with various CMH stakeholders who had recently been involved in a multi-site implementation project. The interviews elicited participant experiences of becoming engaged in the implementation effort. We then compared the interviews data with the experts’ typology to detect consistencies and discrepancies between the two, in order to ascertain strategies’ confirmation in the field and to identify possible refinements relevant to each ORI stage in the CMH context.

### The implementation project

The context for this study was a project that applied a large-scale implementation approach to a cognitive remediation intervention targeting improved employment outcomes in people with a psychiatric disability. The intervention, “Thinking Skills for Work,” complements vocational rehabilitation services, such as supported employment, and has been shown to be effective at improving cognitive and employment outcomes across multiple controlled trials [21–25]. The large-scale implementation approach was piloted (NIDILRR Grant # 90DP0096) across supported employment services in the State of Oregon and included both training and technical assistance over the period of 1 year. All implementation activities were conducted remotely, using technology (e.g., online self-paced training program) to support the roll out of Thinking Skills for Work across multiple agencies simultaneously. The State Director of Supported Employment who served as a project liaison, facilitated the agencies’ recruitment and provided input on training materials prior to launching the implementation activities. Also prior to launching the project, representatives from interested agencies participated in a video conference with the training team, to learn more about the project and clarify expectations so that they would be better able to identify interested and appropriate trainees from their staff.

### Data collection

We conducted 12 retrospective, in-depth interviews, nine of them with staff from the four agencies involved in the pilot implementation project in various roles (see Table 1). Those interviews took 40–60 min. We also interviewed three members of the implementation team, including the intervention’s lead-developer, the training coordinator, and the State Director of Supported Employment. Those interviews took between 60 and 90 min, as they covered the interviewee’s interactions with all participating agencies and across multiple phases of the project. The interviews were conducted by the first author who worked as a research assistant with the implementation team.

**Table 1:** Study participants

Stakeholder group	N	Female	Male	Range of the Years of experience in CMH	Education level	Involved in decision making	Trainee
Implementation Team	3	2	1	15-30	MA, PhD	N/A	N/A
Directors of Clinical / Employment Services	2	0	2	20-40	MA	+	-
Supported Employment Supervisors	4	3	1	6-21	MA	+	+
Supported Employment Providers	3	2	1	10-16	MA, BA	-	+
Total	**12**	**7**	**5**				

Interviews focused on the participants’ experiences during recruitment and preparation for the implementation effort. Participants were asked about the time period from when they first learned about the project to the beginning of the training. We used three different interview guides, one for agency directors and supervisors who were involved in making the decision to join the project, one for staff members who were brought in after the decision to implement the intervention was made, and one for the implementation team members. All participants were asked about their role in the project, their overall experience, and how they were introduced to the project. The directors and supervisors were asked about personal and organizational challenges they encountered, what helped them overcome these challenges, and what influenced their decision to enroll their agency in the project. These interviewees were also asked how and to whom they introduced the project within their organization and how they responded if resistance was encountered. Supplement file number 1 presents the three interview guides.

The providers were asked about their views prior to implementation regarding personal and organizational barriers to taking on the new intervention, what was done to address those concerns, and by whom. The implementation team members were asked about the challenges they had experienced in getting buy-in from different stakeholders and overcoming those challenges. All participants were prompted to describe the strategies used to address attitudes, beliefs, and behaviors towards engaging in the implementation project. Finally, the participants were asked to suggest additional strategies that could have helped them or others to become even more primed, motivated, and prepared for the implementation process**.**

### Procedures

The University Institutional Review Board declared the study to be non-human subject research. The implementation team was recruited by the first author, and the study participants from the agencies were recruited with the help of the state liaison. While informed consent was not required, all participants received information via email about the study goals, expectations, data security measures, and the voluntary nature of their participation. All interviews were conducted through the Zoom video-conferencing platform. The interviews were recorded and transcribed verbatim. The transcripts were de-identified for analysis purposes, using a code number to replace personal and agency identifiers. Participants received a gift card as a token of appreciation for their time and input.

### Analysis

All 12 interviews were analyzed in three steps.

#### Step 1 – Coding interviews for ORI stages

The first author applied a content analysis approach [26] using the qualitative analysis software NVivo 12 to identify the pre-implementation ORI stages each participant went through (not including the implementation team). The coding was informed by behavioral and psychological markers related to each ORI stage consistent with the Transtheoretical Model (see Table 2).

**Table 2:** Behavioral markers of the ORI stages

Stage	Condition	Readiness needs	Expected outcome
ORI-1 Pre-Contemplation	No awareness of the need to change practice or intention to act OR demoralized from having tried changing practice in the past and failed.	Knowledge about the intervention and the change process, exposure, inspiration, explanation of general benefits for the clients and the organization	Willingness to consider, but not to act (interest)
ORI-2 Contemplation	Acknowledge the need for change, open to the change, but expresses concerns about risks, costs, and ambivalence.	Identifying personal benefits of the change, verifying supports, and evaluating the feasibility of the change process	Willingness to become actively involved (motivation)
ORI-3 Preparation	Acceptance and readiness to make small steps towards the change	Developing belief in the success of the process, securing resources	Planning active steps (preparedness)

Adapted from the Transtheoretical Model [19]

#### Step 2 – Coding for ORI strategies

**Fig. 1:**
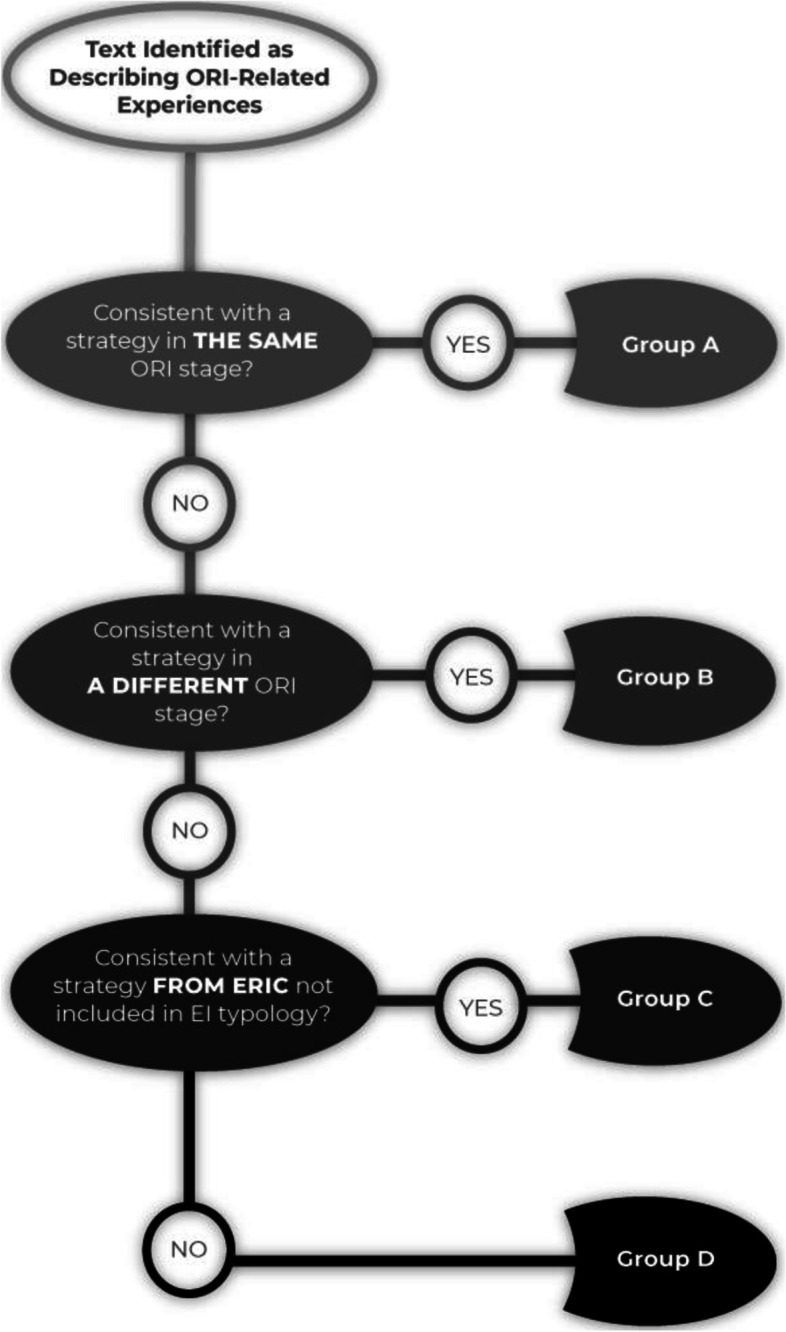
Stepwise confirmation process for the coding of ORI strategies

Two researchers analyzed the text corresponding to each ORI stage. The codebook used for this step consisted of the EI typology developed in our previous study [27] and all other ERIC implementation strategies that the experts considered not relevant to ORI development. The coders developed new codes for strategies mentioned in the interviews that were not included in the ERIC list. For each part of the text identified in Step 1, the coders followed a stepwise coding process (see Fig. 1) using the following questions: (A) Is the text consistent with a strategy assigned to the same ORI stage in the EI typology? (B) If not, is the text consistent with a strategy assigned to a different ORI stage in the EI typology? (C) If not, is the text consistent with a strategy from the ERIC list that was not included in the EI typology? (D) If not, is the text describing another strategy not included in the ERIC list? While a positive response to question A represents consistency with the experts’ classification, the remaining three categories reflect different types of refinements to the typology, based on findings from the field. Each researcher coded the data independently. The researchers met several times after completing the analysis of two to three interviews to develop consensus about their coding and the formulation of new coding categories before moving on to additional interviews.

#### Step 3 – Confirmation of the EI typology

We explored the consistency of implementation strategies reported by the participants with strategies in the corresponding stage of the EI typology. We summarized the findings from Step 2 of the analysis and organized the strategies reported by study participants into four groups: (A) strategies reported in relation to an ORI stage consistent with the same ORI stage as in the EI typology; (B) strategies consistent with a different ORI stage than in the EI typology; (C) strategies consistent with an ERIC implementation strategy not included in the EI typology; (D) new strategies, not included in the ERIC list; and (E) strategies from the EI typology that were not reported in this study. Summaries were developed for each ORI stage. Steps 2 and 3 were reviewed for validation by the second author, who is an implementation expert.

## Results

**Fig. 2:**
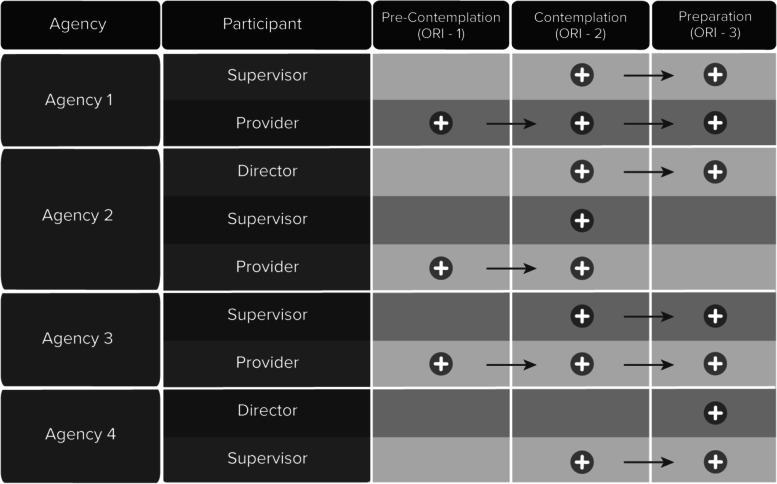
Study participants’ progress through the ORI stages

The participants’ experiences spanned all three ORI stages. However, only providers described experiences relevant to the Pre-contemplation stage, while the initial experiences of most directors and supervisors were related to the Contemplation stage. One agency director was already familiar with the program; therefore, his initial stage was consistent with the Preparation stage. Overall, seven out of the nine field participants reached the Preparation stage, while two from the same agency remained in contemplation (see Fig. 2). In this agency, the supervisor expressed strong resistance to the change and did not develop any motivation to participate. For example, she said “I was there because I was required to.” In contrast, a supervisor from another agency who reached the Preparation stage expressed her motivation to participate in the project by saying, “Looking at the material it was pretty easy for me to figure out what they’re [the researchers] doing and why they’re doing this.” Such examples of motivation to change practice or lack thereof were evident across all field participants. Directors and supervisors reflected more on their ability to engage others in the process. For example, one of the directors described how he engaged the supervisor saying: “I talked about just the time commitment, you know, both for her and for the staff that she was choosing to get trained.” One of the supervisors described engaging other staff members in the project as follows: “We really wanted them to know and to be thinking about clients of theirs that we may be currently serving or have served who could benefit from it.” The level of directors’ involvement in engaging others differed across agencies depending on the dynamics between them and the supervisor and their level of involvement in integrating the project.

Strategies that were described most frequently by participants from all stakeholder groups were “Develop a formal implementation blueprint,” “Assess for readiness and identify barriers and facilitators,” “Identify early adopters,” and “Conduct educational meetings.” Strategies that were not described by any of the participants were mostly related to the Preparation stage of the EI typology such as “Alter patient/consumer fees,” “Change accreditation or membership requirements,” “Change record system,” and “Develop disincentives.”

### Congruence by ORI stage

#### ORI-1, Pre-contemplation

All five implementation strategies (100%) classified as ORI-1 in the EI typology (Group A) were reported by study participants as being used in this stage. They also reported the use of three strategies that the experts assigned to later stages of the typology (Group B; e.g., “Identify and prepare champions”), and two strategies from the ERIC list that were not included in the typology (Group C, e.g., “Develop academic partnership”). Finally, they identified three new strategies (Group D, e.g., “Market the innovation”) not included in the EI or ERIC classifications. Overall, 13 strategies were reported by the participants as relevant for the Pre-contemplation stage. Supplement file 2 presents all study findings.

#### ORI-2, Contemplation

Sixteen of the 18 strategies (89%) included in this stage of the EI typology were reported by study participants (Group A). In addition, seven strategies were reported in this stage that were classified in the EI typology as ORI-3 only (Group B, e.g., “Tailor strategies”). Four strategies from the ERIC list not included in the EI typology were reported by the participants in this stage (Group C, e.g., “Provide ongoing consultation”). Finally, three new strategies described by the participants were identified as relevant to this stage (Group D, e.g., “Recruit a local coordinator”) which were also relevant to ORI-1. Overall, 30 implementation strategies were identified as relevant for the Contemplation stage.

#### ORI-3, Preparation

Twenty of the 38 strategies (53%) assigned to this stage by the experts were also reported by the study participants (Group A). Three strategies assigned by the experts to ORI-2 were reported in this stage (Group B, e.g., “Identify early adopters”). Another three strategies that were not part of the EI typology, but were included in the ERIC list, were reported as relevant to this stage (Group C, e.g., “Mandate change”). Four new strategies were identified as relevant for this stage (Group D, e.g., “Plan for time and space allocation”), with two of them unique to ORI-3 and two also relevant to ORI-1 and ORI-2. Overall, 31 strategies were reported as relevant for the Preparation stage.

### Overall congruence

About two thirds of the EI typology were confirmed by the participants. Out of 48 strategies included in the EI typology, a total of 31 strategies (65%) were reported to be used by participants at the corresponding stage (Group A). The other 17 strategies (35%) were not reported by the participants in this study (Group E). Twelve strategies (25%) from the EI typology were reported in a stage additional to the stage(s) recommended by the experts (Group B). Four of the 25 strategies (16%) from the ERIC list that the experts did not include in the typology were reported by participants (Group C). Finally, five new strategies that were not part of the ERIC list or the EI typology were described by the study participants (Group D). Two of the five new strategies were specific only to the Preparation stage: “Plan for time and space allocation” and “Develop and test technical infrastructure.” The other three strategies were mentioned in relation to all three stages: “Involve high level management,” “Market the innovation,” and “Recruit a local coordinator.” Overall, 57 strategies were reported by study participants.

## Discussion

This study is an important step towards establishing a systematic approach to building ORI across the organization. Confirming the EI typology of associations between implementation strategies and the readiness stages, they address with the actual experiences of individuals implementing an EBP at multiple agencies strengthens the combined structure of the Transtheoretical Model and the ERIC compilation. The study also revealed strategies that need to be further specified in terms of their actors, activities, and purpose and offered new strategies relevant to ORI development that were missing from the ERIC compilation. These findings facilitate the expansion of the EI typology with the addition of unique strategies that were not previously identified by implementation experts. Harnessing the stakeholders’ experiences to refine and expand on the experts’ views grounds the ORI typology in “real life” and reinforces its applicability to the CMH field.

Many of the strategies reported in this study were also deemed relevant to pre-implementation phases in other fields, such as “Identify barriers and facilitators for implementation” or “Identify and prepare champions” [15, 16]. However, this study took another step towards specifying the targeted utilization of those strategies within the pre-implementation phase by confirming their association with the behavioral and psychological markers of the ORI stages. As suggested by Presseau et al. [28] clarifying stakeholders’ behaviors is crucial for designing and measuring the outcomes of implementation interventions.

The EI typology and this study also address a knowledge gap identified specifically in mental health research about the temporality, theoretical justification, and outcomes missing in the reporting of implementation strategies [29]. By tying together strategies and pre-implementation stages based on a behavioral framework, we have added an operational layer to both the ERIC compilation and the Transtheoretical Model’s constructs. The findings further expand the results from a previous exploratory study [30], in which we identified strategies and associated actions reported by stakeholders in the CMH field and organized them within the readiness stages of the Transtheoretical Model. Extending this process to the ERIC strategies provides a broader range of behavioral applications to support ORI development. Further exploration is needed to better understand the unique role of different stakeholder groups in applying these strategies within the context of each stage and to list specific actions that can be used to implement each strategy.

While the quantitative findings are preliminary, the results from this study confirm a large portion of the EI typology and offer several additions to each ORI stage. The congruence level found in ORI-1 and ORI-2 was much higher than the congruence in ORI-3. It is possible that since ORI-3 had 38 strategies identified in the EI typology, compared to only five and 18 strategies in ORI-1 and 2, respectively, the study’s limited sample size was not large enough to capture all of them. The fact that strategies in ORI-1 and ORI-2 are more behavioral and general in nature than the strategies in ORI-3 which are more technical and specific might partly explain why not all strategies in ORI-3 were identified in this context. Larger empirical studies could provide more information about the strategies that were not reported in this project. Some strategies may still be relevant to the CMH field, although they were not applied in this specific implementation project, while some might not be relevant due to structural and administrative characteristics of the field.

In addition, several strategies assigned by the experts to ORI-3 relate to the same topic area and, therefore, could be collapsed into one (e.g., “Develop and organize quality monitoring systems” and “Develop and implement tools for quality monitoring”). If such consolidation had been applied to the EI typology, we might have seen higher consistency with the reports from the field. Further exploration of the strategies identified in the EI typology, but not reported in this study, could help distinguish which of them are still relevant for the field and should be included in a revised typology and which strategies could be consolidated.

The 12 strategies expanded to additional stages beyond their EI classification reflect the complexity of organizational readiness [7] that can be resolved with greater specification of their unique utilization in each stage. For example, the findings show that the experts considered several strategies to serve a practical purpose in ORI-3, but these strategies were also assigned to ORI-2 to support decision-making (e.g., “Make billing easier”). According to the participants, the early utilization of these strategies helped them evaluate the feasibility of the project and impacted their attitudes and beliefs regarding the change. Other strategies were applied across all stages but differed in the main actors in each stage. For example, “Recruit, designate, and train for leadership” was used by the implementation team in ORI-1 to recruit the state-level leadership, who used it to recruit executive-level leadership in ORI-2, leading to the recruitment of team-level leadership in ORI-3. The variations in target audience and purpose show how those strategies support the evolution of organizational readiness and how they gradually engage all levels of the project’s hierarchy to establish a collective sense of readiness [6, 7, 31, 32].

Lastly, some strategies reported by the participants were re-visited multiple times in response to recurring issues (e.g., “Tailor strategies”); the experts assigned these strategies to the implementation, rather than pre-implementation, phase. These strategies tend to be broadly defined and could be applicable throughout the implementation process. The ERIC strategies that were not included in the EI typology, but were reported by the study participants, seem to represent such general activities (e.g., “Conduct ongoing consultation”). These types of overarching strategies, used throughout the implementation process, were also reported by Bunger et al. [15], suggesting that some ERIC strategies are broader and not unique to a specific phase. While the strategies that span multiple stages contribute to the flexibility of the typology, they could also benefit from more specification as to the purpose, actors, and actions relevant to their utilization in each stage [29, 33]. Further confirmation is needed to ensure the relevance of these overarching strategies to ORI development beyond the current study.

The new strategies identified by participants broadened the typology and increased its flexibility. The additional strategies were either missing from the ERIC compilation, and therefore from the EI typology, or they underscored a latent aspect concealed in existing strategies that we thought should be highlighted in the context of ORI development. For example, two additions, “Develop and test technical infrastructure” and “Plan for time and space allocation,” align with revisions suggested by Perry et al. [14], based on a utilization evaluation of the ERIC compilation in multiple agencies providing cardiac prevention.

The overarching utilization of the strategy identified as “Develop and test technical infrastructure” is possibly unique to the implementation process piloted in this study, due to the remote nature of training and supervision conducted, as well as the fact that the EBP requires computer-based cognitive exercises. However, as experiences during the COVID-19 pandemic demonstrate, technology is likely to become more and more central to implementation efforts and should receive more attention in the planning phase, including the involvement of technical staff early in the process.

The other new strategies described by participants offer a variation to existing strategies. These include, “Involve high-level management,” “Market the innovation,” and “Assign a local coordinator.” While it could be argued that these three strategies are already covered under existing ones, we found it critical to specify them in relation to ORI development. For example, a local coordinator’s role in soliciting participation, supporting enrollment, providing feedback on educational materials, and solving problems goes beyond the role suggested in the original ERIC strategy: “Identify and prepare champions.” While the new strategies need to be confirmed in other CMH implementation projects, the fact that multiple stakeholders mentioned them at multiple sites supports their relevance to the field. In addition, most of the new strategies were described in relation to multiple stages, suggesting they need to be further specified to differentiate their application in each stage. Supplement 2 presents all the strategies addressed in this study including the new strategies and the stages they were assigned to.

The initial findings related to the differences across stakeholders and agencies indicate that capacity for ORI may vary by the person’s role in the agency and that individual factors may at times override relevant contextual factors. For example, in one agency the supervisor took the lead on integrating the project into the organization while in another agency the supervisor was extremely passive, and the director took the lead. Since supervisors may further influence their staff in either positive or negative way, our findings suggests that development of ORI among supervisors and higher-level managers may need particular attention and possibly additional strategies to address resistance to change among these levels. More importantly, the findings suggest that middle-level management plays a critical role in an organization reaching the Preparation stage, possibly even more than the high-level management.

The primary limitation of this study is the small number of organizations and participants included. Besides the initial small number of participating agencies in the project, not all project participants agreed to share their experiences and contribute to the study. This limitation might have impacted the variety of strategies we gathered, as well as biased responses. The information collected from multiple participants concerning each agency should have mitigated this gap to some extent. In addition, due to the small sample size, it is not possible to make any inferences about agency characteristics and participants’ usage of certain strategies. Future studies might offer more insights regarding the readiness level and choices of strategies in relation to specific organizational culture and context to provide more guidance on best use of the typology.

Another limitation is the specificity of the project. We restricted ourselves to one implementation project with one EBP to eliminate some of the “noise” that characterizes many implementation studies [34, 35]. The cost of this choice is the limited generalizability of our results even within the CMH field. Consequently, our findings should be interpreted with caution when evaluating their relevance in other implementation projects in the field. In addition, although we conducted the interviews within a year from the beginning of the implementation effort, the retrospective interviews could have jeopardized the accuracy of the participants’ reports. Future studies should collect data in real time during pre-implementation.

The results from this initial field testing of the experts’ typology respond to the knowledge gap concerning ORI enhancement. The high level of congruence between the EI typology and the experiences reported in the field confirms the structure of the stage-based model of ORI development. This study implies that the EI typology needs to be further tested in the field on a larger scale to continue its establishment. The high level of consistency found in ORI-1 and ORI-2 and the additional strategies offered for these two stages add critical knowledge about how positive attitudes and beliefs may be established in the early stages of an implementation project. Further inquiries are particularly necessary to address inconsistencies between the experts’ typology and the findings from the field and clarify their relevance to ORI stages. It is also important to have a better conceptual understanding about the overlap of some strategies across stages. While it is possible that certain strategies will have multiple uses, there is a need to further specify their unique utilization in each stage. Additional validation of the behavioral benchmarks achieved by the strategies in each stage will also increase the applicability of the typology as a guiding tool to help advance members of an organization through the ORI stages. Finally, using evidence collected from the target population to support implementation knowledge is a relatively new approach [36–38]. More research involving stakeholders from the field is needed to establish the final ORI typology.

## Conclusion

This initial field testing provided valuable confirmation of a previously developed EI typology of strategies for ORI development and informed further refinements from the perspective of multiple CMH sites and stakeholders. In addition to the high congruence between the experts and the experiences described by study participants, the study findings also expanded the potential utilization of several strategies to other stages and offered new strategies relevant to ORI development that were not known previously identified by the ERIC project. This confirmation and enrichment of the EI typology align with the original purpose of creating a “bank of strategies” to be utilized according to local context and needs. More operationalization (i.e., actors, dose, specific actions, etc.) is needed to better guide the application of the strategies beyond the readiness stages they address. The strategies that were not reported in this study and the strategies that were added to the typology should be confirmed in other CMH implementation efforts to ensure their relevance beyond this specific project. The ORI typology has demonstrated that it holds promise for a practical and dynamic methodology that can position agencies for effective adoption of EBPs that support people with a psychiatric disability.

## Supplementary Information

**Additional file 1:** InterviewGuides.PDF – The three different interview guides used for this study.

**Additional file 2:** Results.PDF – A comprehensive PDF file presenting the results from the analysis of congruence.

**Additional file 3:** Reporting Checklist.PDF – A reporting checklist intended for qualitative manuscripts.

## Data Availability

The datasets used and/or analyzed during the current study are available from the corresponding author on reasonable request.

## Abbreviations

CMH: Community mental health
EBP: Evidence-based practices
EI: Expert-informed
ERIC: Expert recommendations for implementing change
NIDILRR: National Institute on Disability, Independent Living, and Rehabilitative Research
ORI: Organizational Readiness for Implementation
