# Supplementary material for: Building organizational readiness: initial field testing of an expert-informed typology of implementation strategies
Source: Implement Sci Commun. 2022 Mar 2;3:22. doi: 10.1186/s43058-022-00257-7 (PMC8889398; doi:10.1186/s43058-022-00257-7)
Supplement: Supplementary file 1 — Additional file 1: InterviewGuides.PDF – The three different interview guides used for this study. [file 43058_2022_257_MOESM1_ESM.pdf]

INTERVIEW GUIDE FOR THE IMPLEMENTATION TEAM

Interviewer: **Date:**
Interviewee: **Role:**

The following questions refer to your experience during the introduction of TSW into the four agencies participating in the implementation project.

1. Please tell me about your experience with the TSW initiative.
2. Describe your involvement in the implementation of TSW in your agency starting in March 2019.
    - What was your role?
    - Who supported you during the implementation?
    - Did you have a role in deciding to take on this intervention?
3. When were you brought into the process?
    - Event
    - Time before launch
4. Were there any challenges you encountered in getting buy-in for the process?
    - Different stakeholders
    - Objective limitations vs. attitudes/beliefs
5. What do you think helped each agency develop their readiness / buy-in for the implementation process?
6. Please specify activities you were engaged with or aware of:
    - What was the activity?
    - Who was involved?
    - When was it done?
    - Why was this activity used/chosen?
    - What were the results?
    - **Pre-Contemplation**: Exposure to the added benefits of TSW, Inspiration about TSW.
    - **Contemplation**: Help weigh the pros and cons of taking this initiative, personal and professional benefits from adding TSW to practice, increase confidence in ability to add TSW to practice.
    - **Preparation**: Was there, to your knowledge, a plan to implement TSW in the agency? (if yes) What was it? Who created it? Did someone provide resources (time, money, staff) that made it possible? Did someone help create/understand the workplan for implementation (who, when, how)?
7. Do you have additional thoughts related to readiness development during this project?

INTERVIEW GUIDE FOR PROVIDERS

Interviewer: **Date:**
Participant ID: **Agency:** **Role:**

The following questions refer to your experience during the introduction of TSW into your agency.

1. Please tell me about your experience with the TSW initiative at your agency.
2. Describe your involvement in the implementation of TSW in your agency starting in April 2019.
    - What was your role?
    - Who supported you during the implementation?
    - Did you have a role in deciding to take on this intervention?
    - When were you brought along into the process?
3. How were you brought into this implementation process?
    - Did you choose or was it decided for you?
    - What was done to involve you in the implementation process?
    - How did you feel about adding TSW to your practice before starting the training?
    - Did it ended up being how you imagined it to be?
    - Were there any changes in how you felt about adding TSW to your practice?
    - What made you “get on board”? How much time did it take?
4. Did you experience any personal or organizational barriers to taking on TSW? What was done to overcome them? By whom?
    - **Pre-Contemplation**: Was there anything done to help you understand why this change in practice is needed?
        i. You were exposed to the added benefits of TSW (who, when, how)
        ii. You were inspired about TSW (when, how did it help)
    - **Contemplation**: Was there anything done to help you weigh the pros and cons of taking this initiative?
        i. Were you able to learn about your personal and professional benefits from adding TSW to your practice? (by whom, what did you learn, what was the setting)
        ii. Did someone encouraged you or increased your confidence in your ability to add TSW to your practice (who, when, how)
    - **Preparation**: Was there anything done to help you prepare for the implementation of TSW (including training and embedding it in practice)?
        i. Was there, to your knowledge, a plan to implement TSW in your agency?
        ii. (if yes) What was it? Who created it?
        iii. Were you provided with resources (time, money, staff) that made it possible?
        iv. Did someone help you create/understand the workplan for implementation (who, when, how)?

INTERVIEW GUIDE FOR ADMINISTRATORS

**Interviewer:**	**Date:**	
**Start time:**	**End time:**	**Length:**
**Participant ID:**	**Agency:**	**Role:**

The following questions refer to your experience during the introduction of TSW into your agency.

1. Please tell me about your experience with the TSW initiative at your agency.
2. Describe your involvement in the implementation of TSW in your agency starting in April 2019.
    - What was your role?
    - Who supported you during the implementation?
    - Did you have a role in deciding to take on this intervention?
    - When were you brought along into the process?
3. How were you brought into this implementation process?
    - Did you choose or was it decided for you?
    - What was done to involve you in the implementation process?
    - How did you feel about adding TSW to your agency's practice before the training?
    - Did the implementation process ended up being how you imagined it to be?
    - Were there any changes in how you felt about adding TSW to your practice?
    - What made you "get on board"? How much time did it take?
4. Did you experience any personal or organizational barriers to taking on TSW? What was done to overcome them? By whom?
    - **Pre-Contemplation**: Was there anything done to help you understand why this change in practice is needed? Did you do anything to help others in the agency understand this?
        i. Exposure to the added benefits of TSW (who, when, how)
        ii. Inspiration about TSW (when, how did it help)
    - **Contemplation**: Was there anything done to help you weigh the pros and cons of taking this initiative? Did you do anything to help others in the agency acknowledge the pros?
        i. Learn about personal and professional benefits from adding TSW to practice? (by whom, what did you learn, what was the setting)
        ii. Encourage or increase confidence in ability to add TSW to practice (who, when, how)
    - **Preparation**: Was there anything done to help you prepare for the implementation of TSW (i.e., training and embedding it in practice)? Did you do anything to help others prepare?
        i. Was there, to your knowledge, a plan to implement TSW in your agency?
        ii. (if yes) What was it? Who created it?
        iii. Provide resources (time, money, staff) that made it possible
        iv. Create/understand the workplan for implementation (who, when, how)
