# Supplementary material for: Building organizational readiness: initial field testing of an expert-informed typology of implementation strategies
Source: Implement Sci Commun. 2022 Mar 2;3:22. doi: 10.1186/s43058-022-00257-7 (PMC8889398; doi:10.1186/s43058-022-00257-7)
Supplement: Supplementary file 2 — Additional file 2: Results.PDF – A comprehensive PDF file presenting the results from the analysis of congruence. [file 43058_2022_257_MOESM2_ESM.pdf]

Supplement 2. Study Results - Confirmation of the EI typology by Congruency Group

Strategy	Description	ORI-1 Pre-contemplation	ORI-2 Contemplation	ORI-3 Preparation
Access new funding	Access new or existing money to facilitate the implementation		A	E
Alter incentive / allowance structures	Work to incentivize the adoption and implementation of the clinical innovation		A	A
Alter patient / consumer fees	Create fee structures where patients/consumers pay less for preferred treatments (the clinical innovation) and more for less-preferred treatments			E
Assess for readiness and identify barriers and facilitators	Assess various aspects of an organization to determine its degree of readiness to implement, barriers that may impede implementation, and strengths that can be used in the implementation effort		A	B
Build a coalition	Recruit and cultivate relationships with partners in the implementation effort		A	A
Capture and share local knowledge	Capture local knowledge from implementation sites on how implementers and clinicians made something work in their setting and then share it with other sites		C	
Centralize technical assistance	Develop and use a centralized system to deliver technical assistance focused on implementation issues		B	A
Change accreditation or membership requirements	Strive to alter accreditation standards so that they require or encourage use of the clinical innovation. Work to alter membership organization requirements so that those who want to affiliate with the organization are encouraged or required to use the clinical innovation			E
Change physical structure and equipment	Evaluate current configurations and adapt, as needed, the physical structure and/or equipment (e.g., changing the layout of a room, adding equipment) to best accommodate the targeted innovation			A
Change record system	Change records systems to allow better assessment of implementation or clinical outcomes			E
Conduct educational meetings	Hold meetings targeted toward different stakeholder groups (e.g., providers, administrators, other organizational stakeholders, and community, patient/consumer, and family stakeholders) to teach them about the clinical innovation	A	A	A

Strategy	Description	ORI-1 Pre-contemplation	ORI-2 Contemplation	ORI-3 Preparation
Conduct educational outreach visits	Have a trained person meet with providers in their practice settings to educate providers about the clinical innovation with the intent of changing the provider's practice		A	
Conduct local consensus discussions	Include local providers and other stakeholders in discussions that address whether the chosen problem is important and whether the clinical innovation to address it is appropriate	A	A	
Conduct local needs assessment	Collect and analyze data related to the need for the innovation	A	A	
Create new clinical teams	Change who serves on the clinical team, adding different disciplines and different skills to make it more likely that the clinical innovation is delivered (or is more successfully delivered)			A
Create or change credentialing and/or licensure standards	Create an organization that certifies clinicians in the innovation or encourage an existing organization to do so. Change governmental professional certification or licensure requirements to include delivering the innovation. Work to alter continuing education requirements to shape professional practice toward the innovation			E
Develop a formal implementation blueprint	Develop a formal implementation blueprint that includes all goals and strategies. The blueprint should include the following: High) aim/purpose of the implementation; Medium) scope of the change (e.g., what organizational units are affected); Low) timeframe and milestones; and 4) appropriate performance/progress measures. Use and update this plan to guide the implementation effort over time		B	A
Develop academic partnerships	Partner with a university or academic unit for the purposes of shared training and bringing research skills to an implementation project	C	C	C
Develop an implementation glossary	Develop and distribute a list of terms describing the innovation, implementation, and stakeholders in the organizational change			A
Develop and implement tools for quality monitoring	Develop, test, and introduce into quality-monitoring systems the right input—the appropriate language, protocols, algorithms, standards, and measures (of processes, patient/consumer outcomes,			E

Strategy	Description	ORI-1 Pre-contemplation	ORI-2 Contemplation	ORI-3 Preparation
	and implementation outcomes) that are often specific to the innovation being implemented			
Develop and organize quality monitoring systems	Develop and organize systems and procedures that monitor clinical processes and/or outcomes for the purpose of quality assurance and improvement			E
Develop and test technical infrastructure	Install and test software, hardware and machinery needed for training and the delivery of the intervention. Involve IT early in the process.			D
Develop disincentives	Provide financial disincentives for failure to implement or use the clinical innovations			E
Develop educational materials	Develop and format manuals, toolkits, and other supporting materials in ways that make it easier for stakeholders to learn about the innovation and for clinicians to learn how to deliver the clinical innovation	A		
Develop resource sharing agreements	Develop partnerships with organizations that have resources needed to implement the innovation			A
Distribute educational materials	Distribute educational materials (including guidelines, manuals, and toolkits) in person, by mail, and/or electronically			A
Fund and contract for clinical innovation	Governments and other payers of services issue requests for proposals to deliver the innovation, use contracting processes to motivate providers to deliver the clinical innovation, and develop new funding formulas that make it more likely that providers will deliver the innovation		A	
Identify and prepare champions	Identify and prepare individuals who dedicate themselves to supporting, marketing, and driving through an implementation, overcoming indifference or resistance that the intervention may provoke in an organization	B	A	
Identify early adopters	Identify early adopters at the local site to learn from their experiences with the practice innovation	B	A	B
Inform local opinion leaders	Inform providers identified by colleagues as opinion leaders or "educationally influential" about the clinical innovation in the hopes that they will influence colleagues to adopt it	A	A	

Strategy	Description	ORI-1 Pre-contemplation	ORI-2 Contemplation	ORI-3 Preparation
Involve high-level management	Involve executive managers and other administrators in the implementation effort to ensure their support and consult if any adaptations or problem solving are needed	D	D	D
Involve patients / consumers and family members	Engage or include patients/consumers and families in the implementation effort			E
Make billing easier	Make it easier to bill for the clinical innovation		B	A
Make training dynamic	Vary the information delivery methods to cater to different learning styles and work contexts, and shape the training in the innovation to be interactive		B	E
Mandate change	Have leadership declare the priority of the innovation and their determination to have it implemented	C	C	C
Market the innovation	Present the "Why, what, how" to different target audiences, use local success stories, highlight fit with current practices and resources, and with organizational mission and needs	D	D	D
Model and simulate change	Model or simulate the change that will be implemented prior to implementation		B	A
Obtain formal commitments	Obtain written commitments from key partners that state what they will do to implement the innovation			A
Place innovation on fee for service lists / formularies	Work to place the clinical innovation on lists of actions for which providers can be reimbursed (e.g., a drug is placed on a formulary, a procedure is now reimbursable)		B	A
Plan for time and space allocation	Adjust trainees' workload, secure space, and include implementation activities in weekly schedule			D
Prepare patients / consumers to be active participants	Prepare patients/consumers to be active in their care, to ask questions, and specifically to inquire about care guidelines, the evidence behind clinical decisions, or about available evidence-supported treatments			E
Promote adaptability	Identify the ways a clinical innovation can be tailored to meet local needs and clarify which elements of the innovation must be maintained to preserve fidelity		A	A
Promote network weaving	Identify and build on existing high-quality working relationships and networks within and outside the organization,			A

Strategy	Description	ORI-1 Pre-contemplation	ORI-2 Contemplation	ORI-3 Preparation
	organizational units, teams, etc. to promote information sharing, collaborative problem-solving, and a shared vision/goal related to implementing the innovation			
Provide ongoing consultation	Provide ongoing consultation with one or more experts in the strategies used to support implementing the innovation		C	C
Recruit a local coordinator	Recruit and collaborate with a local leader to help with logistics, identify and engage participants as well as provide insights on facilitators and barriers	D	D	D
Recruit, designate and train for leadership	Recruit, designate, and train leaders for the change effort	B	A	A
Revise professional roles	Shift and revise roles among professionals who provide care, and redesign job characteristics			A
Shadow other experts	Provide ways for key individuals to directly observe experienced people engage with or use the targeted practice change/innovation		E	E
Stage implementatio n scale up	Phase implementation efforts by starting with small pilots or demonstration projects and gradually move to a system wide rollout			E
Tailor strategies	Tailor the implementation strategies to address barriers and leverage facilitators that were identified through earlier data collection		B	A
Use advisory boards and workgroups	Create and engage a formal group of multiple kinds of stakeholders to provide input and advice on implementation efforts and to elicit recommendations for improvements		E	E
Use an implementatio n adviser	Seek guidance from experts in implementation		A	E
Use data experts	Involve, hire, and/or consult experts to inform management on the use of data generated by implementation efforts [ensure fit with organizational and federal regulations]			A
Use data warehousing techniques	Integrate clinical records across facilities and organizations to facilitate implementation across systems			E
Use train the trainer strategies	Train designated clinicians or organizations to train others in the clinical innovation			E

Strategy	Description	ORI-1 Pre-contemplation	ORI-2 Contemplation	ORI-3 Preparation
Visit other sites	Visit sites where a similar implementation effort has been considered successful		A	B
Work with educational institutions	Encourage educational institutions to train clinicians in the innovation			E

Note: A=Full congruence; B=Additional stage; C=From ERIC; D=New; E=Not confirmed
